# Iodine

**Published:** 1907-07

**Authors:** W. C. Abbott

**Affiliations:** Chicago, Ill.


### IODINE.

By W. C. Abbott, M. D., Chicago, Ill.

In The Lancet for Sept. 8, Burnet treats of the therapeutic effects of the iodides. Old as is the use of these agents there is still something to be learned regarding their action.

In aneurism Burnet says no single drug acts so beneficially as potassium iodide. Balfour attributes the effect to a uniform dilation of the arterioles that lowered vascular pressure, the blood being rendered more fluid, and the muscular coat showing hypertrophy in all cases where any success had followed the treatment. Burnet, however, believes that the iodide renders coagulation easy and to that attributes the benefit. He instances the cure of hemophilia by the same remedy as significant. Given by the mouth he finds no effect whatever from iodides, on the pulse-rate of the arterial pressure. Injecting iodipin hypodermically for prolonged periods he detected no effect on the vascular tension or on the heart, but obtained benefit nevertheless, without the gastric disturbance always attending the oral administration of potassium iodide.

Stockman had previously arrived at similar conclusions regarding the iodides given by the mouth. Burnet suggests that the benefit may be due to the elimination of toxins that produce arterial degeneration, and the removal of pathologic deposits from the vessel coats, restoring their natural elasticity. When the malady is syphilitic the action is obvious, but many aneurisms are not specific.

Similar difficulties confront us in explaining the action of iodides in arterisclerosis. Vierordt asserted that iodides alleviate and actually cure this disease, giving them from one to three years. He assured that they promote resolution of the vascular deposits. Huchard said they accelerate peripheral and visceral circulation, activate the nutrition of organs, lower arterial pressure and resolve the sclerosed tissues. Stockman found that potassium iodide given intravenously depressed the heart markedly, small doses producing a transient rise in vascular pressure with slow heart; but this was due to the potash, as sodium iodide had no depressant effect. Here also Burnet attributes the benefit to elimination, the potash salt being largely converted into sodium iodide in the body. But the excretion of the uric acid is lessened by iodides, the acid being retained in the body (Haig).

To be of service in angina pectoris the iodide must be given in doses of 30 to 60 grains a day, for months. Hurchard gave it up to 300 grains a day with benefit. Rolleston says the iodides act here by stimulating thyroid secretion; and Fraenkel, who attributes angina to coronary sclerosis, believes that iodide relieves the spasm of the smaller cardiac vessels and the consequent anemia. He prefers sodium iodide and Burnet coincides with his views.

In dealing with respiratory affections we find firmer ground. In stone-masons' fibrosis he had found the most valuable drugs to be potassium iodide and ichthyol. The former almost always relieved emphysemal dyspnea if given for prolonged periods. The action of iodipin is superior and more durable, as it is more slowly eliminated, and being also more slowly absorbed the effects are more uniform. The iodide is all excreted within 24 hours; iodipin may be detected in the urine many weeks after its use has been stopped. Of the 10 per cent. strength he gives one or two drams in warm milk three times a day. One dram equals 8 grains of potassium iodide. The morning cough and expectoration are relieved by a full dose at bedtime.

In the chronic bronchitis of the elderly he gives iodides in water half on hour after meals, and finds 15 grains at bedtime act like a charm. If hyperchlorhydria is present he adds ammonium carbonate. In true bronchitic asthma he gives 10 to 20 grains every two hours for some time. Haig thinks the relief is due to clearing the blood of uric acid, but Burnet attributes it to an antispasmodic action, through the nervous mechanism.

In tertiary syphilis he strongly advocates iodipin hypodermically in 25 per cent. strength. While the effects are probably explicable as eliminative, iodides have been employed as antitoxic agents, by Tarnier in puerperal fever, Dougard in ague, and in erysipelas by others.

In chronic rheumatism the benifit is due to the action on uric acid. If salicylates are also used they eliminate uric acid while the iodide prevents it combining with an alkali in the blood.

In effusion the iodides act by elimination. In intertitial nephritis potassium iodide acts as a diluretic. Hepatic cirrhosis may improve under this remedy. Metallic poisons are set free by it. In goiter he advises small doses early in the course; in exophthalmic goiter it may be of use in the later stages, when the thyroid shows exhaustion and the heart spurious hypertrophy.

After all, he concludes that we know very little as to the rationale of its action, but have merely formulated some useful working hypotheses. "Our lack of knowledge is largely owing to our neglect of clinical experiment and observation. Even the everyday country practician may discover new uses for old remedies and fresh reasons for their employment, because he is daily working in nature's laboratory—the sickroom."

We need precise observations on calx iodata, which may enable us to say if it is eliminated as quickly as potassium iodide. We know that calx iodata acts more speedily, and in such emergencies as cerebral, ocular or nasal syphilis, where we desire to quickly check the destruction of tissues, we rely on the new preparation, pushing it in full doses to iodism. In general it may be administered grain for grain to replace the iodides. Although the actual quantity of iodine is far less, it makes up by its superior activity. We are of the opinion that it is not eliminated so rapidly, but we want those who are using it to make the tests for iodine in the urine and ascertain positively.

We who advocate the active principles and precise medication, are yet not exclusivists, but stand ready to advise and use any other remedy whenever we believe it to be the best for our case. Why, then, do we so seldom see that old standby, potassium iodide, mentioned in our literature?

For several reasons; This remedy has been employed as calomel formerly was, as a sort of general reliance, to be used for all cases unless something else was specifically indicated. Like the diagnosis of malaria, or uric acid, we fell into the habit of seeing "iodide" everywhere. "When in doubt give iodide," was a common enough state of mind. The habit of precise medication makes against this method of panaceal drugging.

Since Bouchard pointed out the toxic nature of the potash element of the urine, and Loeb and later Meltzer showed the vitalizing properties of soda, we have as a rule substituted the latter base in our prescribing. The immense importance of elimination and depoisoning renders potash inadvisable except for special purposes, which would be difficult to name.

As to the action of mercury in syphilis we need waste no time; and the iodides act speedily and do not remain in the system. Iodoform adds to the iodine effect and alleviates any irritability of the stomach that may be occasioned by the other ingredients. Arsenic iodide is probably the most active of the iodine remedies, and as both elements irritate the eyelids it acts as a safety valve, warning us when the destructive action on normal cells commences. The role of phytolaccin is uncertain, but as a working hypothesis we suggest the following: This remedy powerfully stimulates the absorbents, to carry away the loosened debris and with it the materies morbi of the disease. Mercury and iodine do this also, but possibly if this duty is assumed by the phytolaccin the others may be left free to exert their entire force on the specific elements of this disease. This may explain the indubitable improvement in the effects when phytolaccin is added to other ingredients.

The above dose is given three times a day and gradually increased to seven, or until the irritation of the eyelids indicates toxic action, when the dose is to be slightly lessened until this irritation ceases, and then continued in accordance with the rules for treating syphilis, until all evidence of the disease shall have been absent for three months. After nearly six years' use of this formula, the writer can say that he has not secured as good results from any other way of treating syphilis, and has not known a solitary case to show subsequent evidences of the disease. That all such cases are thus permanently cured is too much to claim with the evidence as yet available. These reasons will show why we, with no prejudice against iodide of potassium, rarely mention or use it in our practice today.

We have spoken above of some uses of calx iodata, which by the promptness and energy of its action takes first place in many instances where the old iodides would have been employed. In asthma and various respiratory affections, where Burnet lauds iodide, calx iodata and a compound of iodine and nuclein have received extensive and successful trials in Chicago, not only in our hands but in those of many who are not specially advocates of active principles.

In the treatment of aneurism veratrine has proved so valuable that it has for the time obscured the slower and less-decided operation of iodide. If we see in the latter merely a means of relaxing vascular tension, we can do this more effectively and regulate it more directly and easily by using veratrine. That iodide promotes coagulation is a new claim and we may possibly do well to add it to the sedative. In fact, as this suggestion is obiously advanced to account for the good effects noted clinically from the use of the iodide, we may employ the latter empirically until it has been determined scientifically just how the good is attained.

In arteriosclerosis the iodide has given place with us to the far more effective and more easily administered arsenic iodide, which in doses of a milligram four times a day replaces the bulky doses of the iodide. We get quicker and better effects, and we believe arsenic favorably affects the nutrition of the heart as well. This applies to the interval treatment of angina pectoris also, word for word. Potash assuredly does the cardiac muscle no good.

We come now to syphilis; and here we have learned to use a combination whose effects are so much more powerful, more enduring and more speedily developed, that we have no use for potassium iodide in any phase of this malady. This combination consists of mercury biniodide, gr. 3.67, iodoform gr. 1-2, arsenic iodide, gr. 1.67, and phytolaccin gr.

